# Association of Perceived Stress with Stressful Life Events, Lifestyle and Sociodemographic Factors: A Large-Scale Community-Based Study Using Logistic Quantile Regression

**DOI:** 10.1155/2012/151865

**Published:** 2012-10-04

**Authors:** Awat Feizi, Roqayeh Aliyari, Hamidreza Roohafza

**Affiliations:** ^1^Department of Biostatistics and Epidemiology, School of Health, Isfahan University of Medical Sciences, Isfahan 8174675731, Iran; ^2^Mental Health Department, Isfahan Cardiovascular Research Center, Isfahan University of Medical Sciences, Isfahan, Iran

## Abstract

*Objective*. The present paper aimed at investigating the association between perceived stress and major life events stressors in Iranian general population. *Methods*. In a cross-sectional large-scale community-based study, 4583 people aged 19 and older, living in Isfahan, Iran, were investigated. Logistic quantile regression was used for modeling perceived stress, measured by GHQ questionnaire, as the bounded outcome (dependent), variable, and as a function of most important stressful life events, as the predictor variables, controlling for major lifestyle and sociodemographic factors. This model provides empirical evidence of the predictors' effects heterogeneity depending on individual location on the distribution of perceived stress. *Results*. The results showed that among four stressful life events, family conflicts and social problems were more correlated with level of perceived stress. Higher levels of education were negatively associated with perceived stress and its coefficients monotonically decrease beyond the 30th percentile. Also, higher levels of physical activity were associated with perception of low levels of stress. The pattern of gender's coefficient over the majority of quantiles implied that females are more affected by stressors. Also high perceived stress was associated with low or middle levels of income. *Conclusions*. The results of current research suggested that in a developing society with high prevalence of stress, interventions targeted toward promoting financial and social equalities, social skills training, and healthy lifestyle may have the potential benefits for large parts of the population, most notably female and lower educated people.

## 1. Introduction

Stressful life events can affect the human body responds through activating the sympathetic nervous system and the hypothalamic-pituitary-adrenal axis, which may in turn affect the cardiovascular, the metabolic, and the immune systems [1–3]. Current clinical and epidemiological studies have emphasized the role of stress as an important risk factor for physical and mental disorders that capture the causes of morbidity and mortality particularly in developed societies and recently in developing countries [4, 5]. Psychological health indicators are important for monitoring and evaluating the health status of communities. Some reasons are as follows: (1) psychological health problems contribute heavily to the total burden of disability in the population, especially within the younger age groups [3]; (2) they are prevailing in developing countries, for instance, approximately 36 percent of the Iranian population, as a young population society, suffers from high stress level [6].

Psychological stresses are also associated with huge increase of mortality in general population, which cannot be wholly explained by unusual causes of death, such as suicide [7, 8]. This excess mortality due to usual causes in people with psychological stresses could be partly explained by the association between psychological stresses and unhealthy lifestyles [9].

Perceived stress is a dynamic multidimensional concept, with a wide spectrum of causative and conducive factors. The perceptions comprise medical, physical, psychological, and psychosocial aspects and are both culturally and socially context dependent [10].

The degree of stress experienced and the ways in which a person reacts to it can be influenced by a various number of factors such as personal characteristics, lifestyle, social support, and appraisal of the stressor(s), life events, and sociodemographic and occupational variables.

It is wellknown that socioeconomic factors are the main determinants of psychological health [11]. In this regard, risk factors that might be involved include behavioral factors and material/structural factors. Educational level creates differences between people in terms of access to information and the level of proficiency in benefiting from new knowledge. Inequalities in income may have extra individual or contextual effects that structure the social environment in ways that affect the health of a population [12, 13]. Gender's effect is another notable factor in which some studies suggested that health outcomes for men and women are differently affected by stress which may be explained by sex differences in vulnerability, stress response, or coping strategies [14–16].

Majority of studies showed that among chronic stressors, life events, economic problems, job insecurity and job conflicts, and social and family problems have highest prevalence [17–21]. There is consistent evidence that the perceived job insecurity has significant adverse effects on psychological morbidity [22–25]. Economic stresses can increase the risk of depression; longitudinal studies approved these findings [12, 20, 26]. The role of social support is protective. Inadequate social support has a direct relationship with a low performance and leads to negative health consequences particularly mental health problems [12, 27, 28].

Most researches on mental health and perceived stress have focused on sources of stress within specific population [29–35]. On the other hand, there is few data about perceived stress and particularly its correlated determinants in Iranian general population. Results of few earlier studies showed that stress was a prevalent disorder among Iranian general population [36, 37]. The sources of stress need to be examined in order to develop strategies for reducing stress and increasing satisfactory among general public. Also, sources of stress may be used as explanatory variables in predicting outcome measures such as mental health [15, 18–20, 38]. This may be helpful in developing interventions directing to improve general public well-being.

Therefore, the aim of the current study was to analyze the association between levels of perceived stress as the dependent variable and life event stressors as the predictor variables, controlling for the impacts of sociodemographic and lifestyle variables in a general population living in Isfahan, Iran, using a comprehensive statistical method, that is, logistic quantile regression.

Previous studies have not had sufficient statistical power to address comprehensively major causes and risk factors affecting the perceived stress among general population. Most of the analysis of stress as the outcome variable has applied conventional regression methods. However, it has been implied that the resulting estimates of various effects on the conditional mean of stress were not necessarily indicative of the size and nature of these effects on the entire of perceived stress distribution. A more complete picture of covariate effects can be provided by estimating a family of conditional quantile functions [39, 40]. 

This modeling framework has been adapted for handling bounded outcome variables as a function of exploratory variables [40]. Bounded outcome variables take on values within a specific range. Such variables despite being extremely frequent in many applications, the inferential applicability of traditional analytical methods cannot be constrained to the values of the outcome within the feasible range [41, 42]. Perceived stress, in current study, was considered as the bounded outcome (i.e., dependent) variable; accordingly, its modeling was done using logistic quantile regression. 

## 2. Materials and Methods

### 2.1. Study Design, Participants, and Sampling Strategy

In this cross-sectional study design, the used data were parts of the information collected in sixth phase of Isfahan Healthy Heart Program (IHHP) in 2009. The sample size was 4583. The study sample was extracted using multistage cluster sampling. Inclusion criteria included aged 19 years and over and Iranian citizenship that at least 6-month residence in the area of the study, that is, living in Isfahan and Najafabad, Central Iranian province, consisting of urban and rural areas. Exclusion criteria were pregnancy, prior history of mental disorders, mental retardation, and physical disability. Written informed consent was obtained from individuals, prior to participating in the study. IHHP study was approved by the Ethics Committee of the Isfahan University of Medical Sciences. Details about the methodology used for the IHHP, including sampling method, survey instruments, data entry, and evaluation of IHHP has been explained in elsewhere [43].

Participants underwent a 45 min home interview by trained health professionals to determine general characteristics, lifestyle behaviors, physical activity, life events stressors, and stress level and to fill out the Food Frequency Questionnaire (FFQ).

### 2.2. Instruments

#### 2.2.1. Perceived Stress

Perceived stress was evaluated using self-administered Goldberg General Health Questionnaire (GHQ-12); the scoring system used in current study was the method (0-0-1-1) resulting in that each participant could obtain score from 0 to 12. Validity and reliability of the Iranian version of GHQ has been investigated [44]. In the present paper perceived stress score was considered as the bounded outcome (dependent) variable.

#### 2.2.2. Stressful Life Events

Stressors were measured using a self-administered stressful life events questionnaire. The questionnaire has 46 items having 11 various dimensions including home life, financial problems, social relation, personal conflicts, job conflicts, educational concerns, job security, loss and separation, sexual life, daily life, and health concerns. According to potential importance of financial problems, job security, social relations, and family conflicts stressors and their generality among all people, we focused on these factors. Financial stress was measured with five items: (1) get in to debt, (2) major financial problems, (3) low income, (4) taking on a mortgage, (5) financial inflation; job security was measured with five items (1) job layoff, (2) long-lasting unemployment, (3) concern about job future, (4) high responsibility job, and (5) low salary; social relations were measured with four items: (1) social discrimination, (2) major social changes, (3) social insecurity, and (4) concern about your future and family conflicts that were measured with seven items: (1) addiction (self or family member), (2) divorce or separation, (3) concern about addiction of a family member, (4) quarrels with spouse; and (5) being accused, (6) legal problems, (7) troubles with children. Each domain was assessed with specific number of items using a five-point response scale (“strongly disagree–strongly agree”). Stressful life events questionnaire has been validated in an Iranian general population [45].

#### 2.2.3. Nutrition Practice

A self-administered 56-item food frequency questionnaire (FFQ) was applied to obtain information on dietary fruit and vegetable intake for each participant. The FFQ was a semiquantitative Willett format questionnaire. Participants reported their frequency of consumption of a given serving of each food item during the previous year on a daily (e.g., bread), weekly (e.g., rice, meat), or monthly (e.g., fish) basis. For our analysis, a global dietary index (GDI), expressing global diet quality, was created by calculating the average of the mean of twenty-nine frequency questions in seven categories. Higher scores on the GDI represent diets higher in total fat, saturated fat and cholesterol (unhealthy diet). More details were depicted elsewhere [46].This questionnaire was standardized for 2000 sample using specific Iranian nutrition software analysis. It has been validated for an Iranian general population[47].

#### 2.2.4. Physical Activity

Daily physical activity was determined based on four types of physical activity, that is, leisure time activity, work activity, commuting activity, and home activity. Physical activity in each four types was assessed according to the frequency of most common activities of Iranian population (number of sessions per day) and average duration (hours and minutes per session). The intensity of physical activity in all four types was expressed in metabolic equivalents (METs). One MET is equal to 3.5 mL/kg/min O_2_ uptake. This questionnaire has appropriate validity and reliability [37].

### 2.3. Other Variables Included in Study

Age, gender, marital status (married/single), educational level (illiterate, 1–8, 9–12, and >12 years of formal schooling), type of job, income level, place of residence, and smoking habits were other variables that included in statistical modeling. 

### 2.4. Statistical Analysis

Logistic quantile regression (LQR) was used as the main statistical method for modeling the relation of independent variables including stressful life events (economic problems, job insecurity, social problems, and family conflicts) adjusted by lifestyle factors (physical activity, performance nutrition and smoking) and demographic variables, that is, age, gender, marital status, education level, type of job, and place of residence with perceived stress as the bounded response variable.

Between-group comparisons were conducted using one-way ANOVA and Students *t*-test. The simple association of dependent and independent variables was investigated using Pearsons moment correlation coefficient. All statistical analysis was conducted using R Free Statistical Software version 2.13.1.

### 2.5. Theoretical Background for Quantile Logistic Regression

Ordinary least-squares regression models the relationship between one or more covariates *X* and the conditional mean of the response variable *Y* given *X* = *x*. Quantile regression, which was introduced by Koenker [39], extends the regression model to conditional quantiles of the response variable [39]. Quantile regression is particularly useful when the rate of change in the conditional quantile, expressed by the regression coefficients, depends on the quantile. The main advantage of quantile regression over least-squares regression is its flexibility for modeling data with heterogeneous conditional distributions. Quantile regression provides a complete picture of the covariate effect when a set of percentiles is modeled, and it makes no distributional assumption about the error term in the model. Quantile regression, recently, became popular in biomedical sciences [48, 49].

Quantile regression can be applied to the analysis of continuous bounded outcomes. Bounded outcome scores are measurements that are restricted to a finite interval, which can be closed, open, or half closed. Examples of bounded outcome scores can be found in many medical disciplines. For instance “compliance” can be defined as the proportion of days that patients correctly take their drug. Another example is the Barthel index (Mahoney and Barthel, 1965) which is an activities of daily living scale that takes values from 0 (death or completely immobilized) to 100 (able to perform all daily activities independently). This scale is often used in stroke trials to measure the recovery of a patient after an acute stroke. Finally, in pain and pain-relief studies, a visual analog score (VAS) is used to measure the psychological state of the subject [41].

Frequency distributions of bounded outcomes may assume a variety of shapes including unimodal, U-shape, and J-shape. Accordingly, the analysis of such variables needs specific analytical methods [41, 42]. Lesaffre et al. explored the use of the logistic transformation to a normal variable whose location and scale may depend on covariates [41]. Their approach can be applied to continuous as well as discrete, or coarsened, outcomes defined on some bounded interval. The theoretical backgrounds of logistic quantile regression for the analysis of bounded outcomes are as follows.

First consider a logistic transformation *z* = *α* + *β*log⁡⁡((*Y* − *a*)/(*b* − *Y*)) where *α*, *β*, *a*, *bεR*, and *Y* is a score on the interval (*a*, *b*). The aim of this transformation was to achieve standard normality [50]. Here we take *α* = 0 and *β* = 1 and assume that the logistic transformation achieves a normal density *N*(*μ*, *σ* ^2^). In general, when *Z* has density *f*(*z*) ≡ *f*(*z*; ***θ***), then *Y* has density *g*(*y*) ≡ *g*(*y*; *θ*) = *f*(logit(*u*))1/*y*(1 − *y*), where logit(*u*) = log⁡(*y*/1 − *y*). When *Z* ~ *N*(*μ*, *σ* ^2^), then *Y* has an LN distribution, denoted as LN(*μ*, *σ* ^2^) and ***θ*** ^*T*^ = (*μ*, *σ* ^2^).

If we consider *Y* as a bounded outcome variable from below and above to known constant, a and b and a set of *k* covariates {*x*_1_, *x*_2_, …, *x*_*k*_}, the conditional *p*-th quantile of *y* gives the set of covariates as *Q*_*p*_(*y*), where *p* is a proportion between zero and one, resulting in a fixed set of parameters *β*_*p*_ = {*β*_*p*,0_, *β*_*p*,1_,…, *β*_*p*,*k*_} for any quantile *p*. Using a known nondecreasing link function such as *g* from the interval (*a*, *b*) to real line, for example, logit link, we can express *Q*_*p*_(*y*) as a function of covariates (**X** *s*) as follows:

(1) $$\begin{array}{c} g\left\{Q_{p}\left(y\right)\right\}=\beta_{p,0}+\beta_{p,1}x_{1}+\cdots +\beta_{p,k}x_{k}, \end{array}$$

(2) $$\begin{array}{c} g\left(y\right)=\text{logit}\left(y\right)=\log\left(\frac{y-a}{b-y}\right), \end{array}$$

(3) $$\begin{array}{c} Q_{p}\left(y\right)=\frac{\exp\left(\beta_{p,0}+\beta_{p,1}x_{1}+\cdots ,\beta_{p,k}x_{k}\right)b+a}{\exp\left(\beta_{p,0}+\beta_{p,1}x_{1}+\cdots ,\beta_{p,k}x_{k}\right)b+1}. \end{array}$$

When the sample data are available, an estimate for the regression coefficient can be obtained via quantile regression trough regressing the transformed outcome *g*(*y*) on **x**. Once estimates for the regression coefficients *β*_*p*_ are obtained, inference on *Q*_*p*_(*y*) can then be made through the inverse transform in (3). This is possible because quantiles are invariant to monotone transformations, that is, *Q*_*g*(*y*)_(*p*) = *g*{*Q*_*p*_(*y*)}.

## 3. Results

In this study, 4583 subjects aged over 19 years and over have participated. The mean (SD) age was 39.03 (5.2 years); 51% of them were women, 77.7% were married, and 82.3% of the participants lived in city. Majority of the participants were in medium level of economic status. Complete information about the sociodemographic characteristics of participants is shown in Table 1. 

Table 1 shows the mean (SD) scores of the stressful life events and scores of physical activity and nutritional practice. There was statistically significant positive association between the score of perceived stress and four dimensions of stressful life events and GDI (as the index of nutritional practice, the higher the GDI score the lower healthy nutrition practice). As can be seen from Table 1, mean perceived stress score was statistically different among people with different levels of income (*P* < 0.0001), age groups (*P* < 0.05), educational attainment levels (*P* < 0.0001), and male and female (*P* < 0.0001); however, it was not different between smoker and nonsmoker and married and single people. Also, people who lived in urban were not different from those who lived in rural districts in terms of levels of perceived stress.

A more complete and reliable picture of covariate associations can be provided by estimating a family of conditional quantile functions.

We considered ten quantiles, that is, *P* = {0.15,0.30,0.40,0.50,0.60,0.70,0.80,0.85,0.90,0.95}, and the estimated regression parameters for each quantile are reported in Table 2. The interpretation of the regression coefficients is analogous to the interpretation of the coefficients of a logistic regression for binary outcomes. For example, exp⁡(*β*_0.5,1_) = OR = 1.28 (95%CI: 1.12–1.46) represents the odds ratio of median score in females versus males. Also, for each continuous covariate, these point estimates may be interpreted as the impact of a one-unit change of the covariate on the specific quantile of perceived stress score holding other covariates fixed. For example, when family conflicts increases by one unit, the 0.15th quantile of the logit of perceived stress is estimated to increase by 1.14 units whereas the 0.90th quantile by 1.31. Comparing the covariate coefficients of stressor life events shows that the family conflicts consistently over all considered quantiles seems to be more associated factor on level of perceived stress and social problems; job insecurity and financial problems are in other orders of importance.

As can be seen from Table 1, people in different education levels differently affected by stressors (*P* < 0.0001). Education was negatively (OR < 1, see Table 2) associated with perceived stress score. Table 2 shows that the association of educational attainment levels over the whole range of the outcome distribution. People who were in lower level of education more affected by stressors. However, people who were at 9–12 and >12 of educational attainments, at higher quantiles, tend to be similarly affected by stressors but less than illiterate people.

The disparity between level of perceived stress of people who were in different categories of employment and living in different places of residence was not substantial, particularly at the left tail of the distribution.

**Figure 1:**
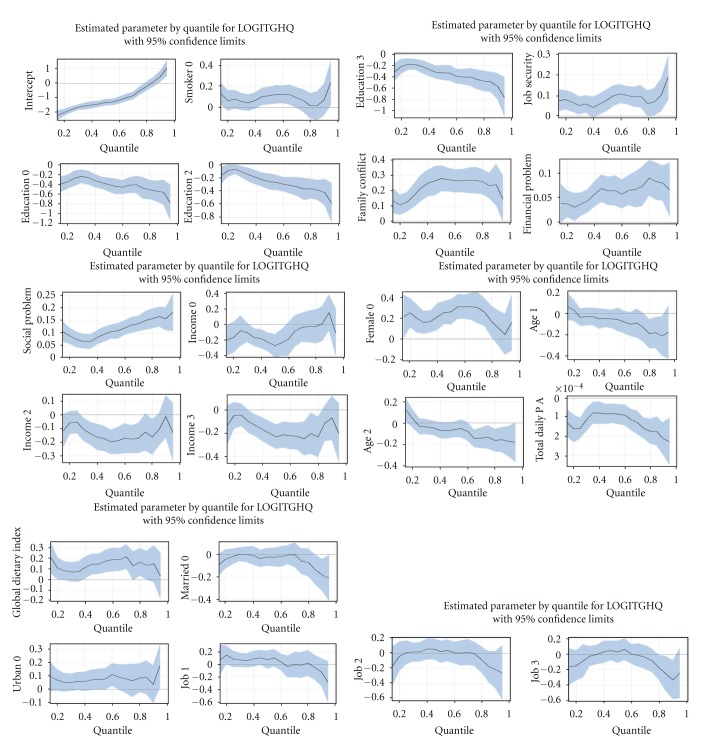
Estimates (solid lines) and the 95 % confidence bands for the regression coefficients associated with continues and each levels of categorical covariates for considered quantiles of perceived stress, *p* = 0.150.30,0.40,…, 0.95. [Smoking behavior; Smoker0 = smoker], [Marital status: married0 = married], [Place of residence: urban0 = urban], [Income levels: income0 = income >500, income2 = income 100–300, income3 = income 300–500], [Age categories: age1 = age>50 years, age2 = age: 30–50], [Educational levels:education0 = education >12 years, education2 = 1–8,education3 = 9–12], [Job type: job1 = housewife, job2 = manual, job3 = non-manual], [Physical activity: total daily P.A1 = > q66,total daily P.A2 = q33–q66].

Higher scores on the GDI scale indicated a greater degree of perceived stress in the study's participants; however, no monotone relation was found (see Figure 1).

Physical activity was categorized to tertiles; it was associated with level of perceived stress in which the participants with high level of physical activity had lower odds of experiencing higher stress level compared to people in moderate level of physical activity.

No clear association was found between type of employment and level of perceived stress. Also area of residence, although urban people experienced more stress, was not a predictor for perceived stress. 

Figure 1 illustrates the estimated conditional distributions of perceived stress, giving all considered predictors. The *x*-axis of each graph shows the quantile levels, and the *y*-axis shows the corresponding perceived stress score. In each panel, the relation of each predictor maintaining the others at constant levels was considered which enabled us to study the impact of that predictor on the entire distribution of outcome variable.

## 4. Discussion and Conclusions

Based on the findings of the present study, controlling for the association of lifestyle factors (smoking, nutrition practice, and physical activity) and demographic characteristics (place of residence, gender, age, income levels, education levels, occupation, and marital status), it was observed that stressful life events including family problems, job insecurity, financial problems, and social relations directly were associated to level of perceived stress. Among the proposed stressful life events, family and social problems had more notable relation with stress perception. This may be related to different Iranian cultural aspects that people are more sensitive to familial and social (engagement) relationships. In accordance with our study, in Sapr ÜNER and colleagues' study on student subjects, direct relation was observed between mental health which was measured by the GHQ-12 and stressful life events including lack of positive events during the past year (OR = 1.32), emotional violence (OR = 1.65), a poor relationship with father and mother (OR = 1.57 versus 1.7, resp.), and poor relationship with partner (OR = 2.29) [22]. Also, some other previous studies have found strong association between psychosocial stressors and mental disorders [32]. In a longitudinal study, Stanfeld et al. studied 7977 individuals and concluded that the higher score of stress was correlated to low social support, in men (OR = 1.31) and women (OR = 1.17) while controlling for stress score at the baseline, age, and place of work [23].

Stress-related side effects of job insecurity and other sources of job stress and career dissatisfaction on people's mental health and other important aspects of their overall well-being have been investigated in previous studies [51]. Previous studies suggested that fulfilling one's need for security and stability may be regarded as more important than the job itself, in which one's overall engagement may be impacted or lowered until one's basic need for security or psychological safety has been satisfied [24, 52].

We found an independent association of financial conflicts with reported levels of perceived stress after adjusting for potentially confounding individual variables; however, its magnitude compared to other stressors was smaller. In line with our research, earlier studies showed that the experienced financial problems concurrently predicted psychological distress [53].

Although in current research being a smoker had positive impact on more perception of stress, it was not statistically significant. Majority of previous studies that studied the relation between stress and smoking have predominantly shown that smokers report higher stress levels than nonsmokers; according to the observation of higher stress in smokers versus nonsmokers, it was concluded that smoking causes stress [54–57]. 

For both men and women, high levels of perceived stress were associated with higher GDI score. In accordance with our study, several studies have obtained similar conclusions [57–59].

Our results in line with Debbie et al. showed that the high level of physical activities was associated with perceived stress reversely [30]. As can be seen from Table 2, low or moderate level of physical activities weakly associated with experiencing low levels of perception of stress. Previous studies on the relationships between stress and physical activity have produced mixed results. In some of these studies, no relationship was approved and some of them concluded that chronic physical activity reduces stress and improves its symptoms [60–62].

Some studies have suggested that the effects of stress on health outcomes are different for men and women, which may be explained by sex differences in vulnerability, stress response, or coping strategies [14, 16, 63]. Compared with women, men seem to respond to stress with greater reactivity of the hypothalamic-pituitary-adrenal axis, which may partly explain the observed sex differences [64].

In current research, it was observed that the married people more affected by stressors than singles particularly over the right tail of distribution of perceived stress; however, marital status was not significantly correlated with stress perception in majority of quantiles. Such a result can be justified by the fact that married people are generally more concerned about their situations because of their responsibilities toward the family. 

According to values of regression coefficients, it can be inferred that the income level was one of the stronger predictors of perceived stress particularly over the less than 80th quantile. The association of income level with perceived stress was most pronounced among those whose incomes were in (300–500$) and >=500$ (i.e., middle and high income levels, resp.). Our findings support the conclusions of some earlier studies [13, 65, 66] and are not consistent with those that supporting the limited resources have a direct negative impact on quality of life and health [67–69].

In the current study, indirect association was observed between education levels and perceived stress. This means that there was a positive relation between high education level and adaptive coping strategies (less affecting by stressors) and a negative relation between low education level and maladaptive coping strategies (more affecting by stressors). For instance, the odds of perceived stress for people with academics attainment at 15th quantile was 0.34 (0.16 and 0.27 for people were in 1–8 and 9–12 years of education, resp.) times less than illiterates while at 95th quantile it was 0.52 (0.31 and 0.38 for people were in 1–8 and 9–12 years of education, resp.), resulting that the individual adaption are affected by education level, in which higher educational qualifications had notable positive role in more stressful situations. Educational level creates differences between people in terms of access to information and the level of proficiency in benefiting from new knowledge [12]. Accordingly, a lower education placed people at a disadvantaged position for majority of the stressors (i.e., financial stress, worse perceived health status, and psychological distress) and resources (i.e., perceived life control, social support, and social cohesion) [68]. Majority of the previous research on the relationships between perceived stress and education level have produced consistent results and supported our findings [33, 68].

Regarding to the relation of age with perceived stress, present study showed middle-aged (30–50 yrs) and elderly people similarly less affected by stressors than young people, although majority of the estimated coefficients over whole distribution of outcome variable were not statistically significant. The reported relation of perceived stress and age in most recent previous studies [6, 68] was in contrast with our findings. This difference may be attributed to socioeconomic status of Iranian society that the young people more faced with high levels of stress due to financial problems and job insecurity. 

### 4.1. Study Strength and Limitations

Strengths of the current study included a large community-based sample and assessment of the relations of multiple stressors that were not considered in earlier studies and most important life style behavior variables, that is, food intake, physical activity, and tobacco use using comprehensive statistical method. Limitations included cross-sectional analysis, in which causal inferences cannot be made. Another limitation was that the dependent variable and majority of behavior measures were subjectively assessed daily and monthly; accordingly, they may be subjected to social preference or memory bias. Other limitation of this study was the ignoring of mediator role of some variables such as health behaviors and educational level that influence people's level of stressors and resources; accordingly, developing a structural equations modeling in this context could be considered as an effective approach. 

The present paper was an M.S. thesis in biostatistics at School of Health, Isfahan University of Medical Sciences, project number: 390248.

## Figures and Tables

**Table 1:** Descriptive characteristics of study's participants and mean (±SD) of perceived stress in each level of predictor variables.

Variables	r*	Mean ± SD	*P* value
Family conflict	0.225	0.29 ± 0.53	<0.0001
Financial problem	0.211	1.67 ± 1.36	<0.0001
Social problem	0.222	1.06 ± 1.21	<0.0001
Job insecurity	0.165	1.09 ± 0.89	<0.0001
Global dietary index (GDI)	0.077	0.82 ± 0.30	<0.0001
Total daily physical activities	−0.086	808.6 ± 621.7	<0.0001

Categories	Number (percent)	Mean ± SD	*P* value**

Age (years)			
19–29	1544 (33.7%)	3.41 ± 2.32	0.026
30–50	2018 (44.1%)	3.33 ± 2.20	
>50	1016 (22.2%)	3.57 ± 2.34	
Job			
Housewife	2005 (43.7%)	3.40 ± 2.27	<0.0001
Manual	969 (21.1%)	3.20 ± 2.17	
Nonmanual	860 (18.8%)	2.86 ± 1.94	
Retired or jobless	694 (15. 1%)	3.76 ± 2.39	
Place of residence			
Urban	3774 (82.3%)	3.41 ± 2.27	0.965
Rural	809 (17.7%)	3.41 ± 2.20	
Marital status			
Married	3563 (77.7%)	3.39 ± 2.25	0.235
Single	1014 (22.1%)	3.49 ± 2.36	
Sex			
Female	2339 (51%)	3.72 ± 2.37	<0.0001
Male	2244 (49%)	3.09 ± 2.13	
Income			
<100$	669 (14.6%)	3.17 ± 2.53	<0.0001
100–300	2585 (56.4%)	3.41 ± 2.27	
300–500	946 (20.6%)	3.02 ± 2.00	
>500	362 (7.9%)	2.95 ± 2.08	
Education (years)			
Illiterate	559 (12.2%)	4.22 ± 2.75	<0.0001
1–8	1837 (40.1%)	3.48 ± 2.24	
9–12	1358 (29.6%)	3.26 ± 2.20	
>12	813 (17.7%)	3.04 ± 2.07	
Smoking			
Current and passive smoker	843 (18.6%)	3.45 ± 2.33	0.547
Nonsmoker	3731 (81.4%)	3.40 ± 2.26	

*r: Pearsons correlation coefficient.

**Based on two independent samples *t*-test or one-way ANOVA.

**Table 2:** Estimated logistic quantile regression coefficients (OR (95%CI)) for perceived stress.

Variables/quantile	q15	q30	q40	q50	q60	q70	q80	q85	q90	q95
Family conflicts	1.14* (1.04–1.25)	1.19* (1.09–1.29)	1.29* (1.18–1.42)	1.33* (1.22–1.45)	1.30* (1.21–1.40)	1.32* (1.21–1.44)	1.29* (1.20–1.39)	1.25* (1.15–1.37)	1.31* (1.16–1.48)	1.14 (0.95–1.37)
Financial problem	1.05* (1.01–1.09)	1.03* (1.0–1.06)	1.06* (1.03–1.09)	1.07* (1.03–1.1)	1.05* (1.02–1.09)	1.06* (1.02–1.10)	1.08* (1.04–1.13)	1.09* (1.05–1.14)	1.08* (1.03–1.14)	1.06 (0.99–1.14)
Social problem	1.1* (1.05–1.15)	1.07* (1.02–1.11)	1.08* (1.05–1.12)	1.11* (1.08–1.15)	1.14* (1.1–1.17)	1.15* (1.11–1.19)	1.17* (1.12–1.22)	1.19* (1.14–1.24)	1.15* (1.10–1.21)	1.19* (1.10–1.28)
Job insecurity	1.08* (1.02–1.15)	1.05 (1.0–1.10)	1.04 (0.99–1.1)	1.08* (1.01–1.14)	1.11* (1.05–1.17)	1.11* (1.05–1.16)	1.07* (1.01–1.14)	1.09* (1.02–1.16)	1.11* (1.02–1.21)	1.20* (1.06–1.36)
Global dietary index (GDI)	1.18* (1.01–1.37)	1.07 (0.97–1.17)	1.11* (1.0–1.24)	1.15* (1.04–1.27)	1.19* (1.06–1.34)	1.23* (1.09–1.40)	1.19* (1.03–1.36)	1.13 (0.98–1.31)	1.15 (0.96–1.38)	1.04 (0.76–1.42)
Age (19–29 years: reference category)										
30–50	1.1 (0.97–1.24)	0.97 (0.9–1.04)	0.94 (0.87–1.02)	0.95 (0.87–1.04)	0.92 (0.84–1.01)	0.89* (0.82–0.98)	0.86* (0.76–0.96)	0.83* (0.73–0.94)	0.85* (0.74–0.98)	0.83 (0.68–1.02)
>50	1.06 (0.91–1.23)	0.98 (0.9–1.07)	0.95 (0.86–1.05)	0.96 (0.87–1.06)	0.93 (0.83–1.04)	0.92 (0.80–1.06)	0.87 (0.74–1.01)	0.83* (0.71–0.97)	0.82* (0.69–0.97)	0.84 (0.66–1.07)
Total daily physical activity (<*Q*_33_: first tertile: reference category)										
*Q*_33_–*Q*_66_ (second tertile)	0.88* (0.77–0.99)	0.97 (0.92–1.04)	0.98 (0.91–1.05)	0.98 (0.90–1.07)	0.98 (0.90–1.06)	0.93 (0.84–1.04)	0.90* (0.81–1.0)	0.95 (0.83–1.08)	0.88 (0.75–1.04)	0.89 (0.76–1.05)
>*Q*_66_ (third tertile)	0.79* (0.70–0.89)	0.90* (0.82–0.99)	0.93 (0.86–1.0)	0.93 (0.85–1.02)	0.94 (0.86–1.03)	0.86* (0.77–0.96)	0.83* (0.73–0.94)	0.83* (0.72–0.95)	0.77* (0.65–0.90)	0.77* (0.62–0.96)
Job (housewife: reference category)										
Manual	0.8 (0.64–1.0)	0.95 (0.85–1.06)	0.94 (0.83–1.07)	0.92 (0.82–1.04)	0.94 (0.81–1.08)	0.99 (0.83–1.17)	0.87 (0.71–1.06)	0.86 (0.71–1.03)	0.89 (0.72–1.09)	1.04 (0.76–1.43)
Nonmanual	0.81* (0.66–0.99)	0.92 (0.80–1.05)	0.93 (0.84–1.04)	0.93 (0.83–1.03)	0.94 (0.84–1.06)	0.94 (0.82–1.09)	0.82* (0.68–0.98)	0.83* (0.70–0.98)	0.83 (0.68–1.02)	1.05 (0.80–1.38)
Retired and workless	0.89 (0.69–1.14)	0.93 (0.82–1.05)	0.90 (0.78–1.04)	0.93 (0.8–1.1)	0.94 (0.80–1.10)	0.97 (0.81–1.17)	0.98 (0.80–1.20)	1.04 (0.85–1.26)	1.13 (0.90–1.40)	1.32 (0.94–1.86)
Urban (versus rural)	1.09 (0.97–1.22)	1.06 (0.98–1.14)	1.05 (0.96–1.14)	1.08 (0.98–1.18)	1.09 (0.99–1.21)	1.08 (0.96–1.23)	1.08 (0.97–1.21)	1.11 (0.97–1.27)	1.05 (0.92–1.21)	1.22 (0.99–1.50)
Married (versus single)	0.92 (0.84–1.01)	1 (0.93–1.07)	1 (0.92–1.08)	0.97 (0.89–1.06)	0.99 (0.90–1.08)	1 (0.90–1.10)	0.94 (0.82–1.07)	0.86* (0.74–0.99)	0.85 (0.73–1.0)	0.8* (0.65–0.97)
Female (versus male)	1.30* (1.04–1.61)	1.18* (1.03–1.36)	1.25* (1.09–1.42)	1.28* (1.12–1.46)	1.37* (1.20–1.57)	1.36* (1.17–1.57)	1.19* (1.02–1.04)	1.12 (0.93–1.35)	1.07 (0.83–1.36)	1.17 (0.82–1.66)
Income (<100$: reference category)										
100–300	0.90 (0.80–1.02)	0.90* (0.82–0.99)	0.83* (0.74–0.94)	0.81* (0.74–0.89)	0.84* (0.76–0.92)	0.83* (0.73–0.95)	0.85* (0.75–0.96)	0.89 (0.79–1.0)	0.97 (0.82–1.13)	0.88 (0.66–1.17)
300–500	0.90 (0.77–1.06)	0.90* (0.81–1.0)	0.83* (0.73–0.95)	0.78* (0.70–0.88)	0.79* (0.70–0.90)	0.76* (0.65–0.89)	0.81* (0.69–0.95)	0.87 (0.74–1.03)	0.93 (0.77–1.13)	0.8 (0.58–1.09)
>500	0.82 (0.67–1.0)	0.88 (0.75–1.03)	0.81* (0.69–0.95)	0.75* (0.63–0.89)	0.82* (0.67–0.99)	0.93 (0.76–0.93)	0.93 (0.75–1.15)	0.97 (0.75–1.24)	1.11 (0.88–1.39)	0.87 (0.63–1.22)
Education (illiterate-years: reference category)										
1–8	0.84* (0.72–0.98)	0.91 (0.81–1.04)	0.83* (0.73–0.95)	0.76* (0.67–0.88)	0.75* (0.67–0.84)	0.70* (0.61–0.81)	0.68* (0.58–0.81)	0.69* (0.57–0.82)	0.65* (0.51–0.82)	0.58* (0.40–0.85)
9–12	0.73* (0.61–0.87)	0.86* (0.74–1.0)	0.78* (0.67–0.90)	0.72* (0.61–0.84)	0.68* (0.59–0.78)	0.66* (0.57–0.78)	0.62* (0.51–0.74)	0.62* (0.50–0.76)	0.58* (0.44–0.76)	0.48* (0.31–0.74)
>12	0.66* (0.54–0.81)	0.81* (0.68–0.97)	0.74* (0.64–0.87)	0.68* (0.57–0.80)	0.65* (0.57–0.75)	0.66* (0.56–0.78)	0.61* (0.50–0.74)	0.59* (0.47–0.74)	0.58* (0.43–0.77)	0.48* (0.32–0.73)
Smoker (versus nonsmoker)	1.1 (0.98–1.25)	1.06 (0.96–1.17)	1.08 (1.0–1.17)	1.11* (1.02–1.22)	1.13* (1.02–1.25)	1.08 (0.97–1.21)	1.02 (0.89–1.16)	1.01 (0.85–1.19)	1.06 (0.86–1.30)	1.22 (0.92–1.60)
Constant	0.13* (0.09–0.17)	0.20* (0.16–0.24)	0.25* (0.19–0.31)	0.29* (0.24–0.36)	0.33* (0.26–0.41)	0.43* (0.34–0.55)	0.80 (0.58–1.10)	1.01 (0.73–1.41)	1.34 (0.85–2.10)	2.01* (1.08–3.73)

*Indicating statistically significant.
